# Exploring the Molecular Adaptations, Benefits, and Future Direction of Exercise Training: Updated Insights into Cardiovascular Health

**DOI:** 10.3390/jfmk9030131

**Published:** 2024-07-26

**Authors:** Michael F. Mendoza, Nina M. Suan, Carl J. Lavie

**Affiliations:** 1 The Gayle and Tom Benson Cancer Center, Ochsner Clinic Foundation, New Orleans, LA 70121, USA; michael.mendoza@ochsner.org; 2 Faculty of Medicine and Surgery, University of Santo Tomas, Metro Manila 1008, Philippines; nnsuan@gmail.com; 3 Department of Cardiology, Ochsner Clinic Foundation, New Orleans, LA 70121, USA; 4 Ochsner Clinical School, The University of Queensland Medical School, New Orleans, LA 70121, USA

**Keywords:** exercise, physical activity, cardiovascular disease, cardiorespiratory fitness, resistance exercise training, aerobic exercise training

## Abstract

This review emphasizes the globally accepted physical activity guidelines and explores the various molecular adaptations that occur with continuous exercise. It is essential to highlight the critical roles of cardiorespiratory fitness, muscular strength, and muscle mass in reducing mortality and enhancing quality of life. It has been shown in various studies that there are certainly substantial reductions in cardiovascular and all-cause mortality among individuals with high cardiorespiratory fitness levels. Resistance training is also examined, which, likewise, reveals significant mortality benefits, even with minimal weekly engagement. When delving into the molecular mechanisms, it is apparent that exercise training favorably influences certain cardiovascular conditions, mostly owing to its effect on enhanced lipid metabolism, improvement in glucose regulation, ability to modulate inflammation and oxidative processes, and induction of other cardioprotective effects like improved sympathetic tone and left ventricular remodeling. Cardiovascular diseases and malignancy also share the same risk factors, which explains why exercise can also mitigate the risk of developing many types of cancers. But despite these advancements in research, cardiovascular diseases continue to be prevalent, which may suggest the need to devise other means of promoting physical activity involvement. These approaches may include a greater emphasis on the societal benefits of increased exercise adherence, facilitated by community involvement and technological advancements in fitness tracking devices. We conclude that the future directions for exercise research should emphasize the need for personalized or tailored exercise programs to make it more engaging, accessible, and inclusive for a diverse set of people.

## 1. Introduction

The American Heart Association (AHA) succinctly asks, “Are you fitting in at least 150 min (2.5 h) of heart-pumping physical activity per week?”. This is an abbreviated question based on the Physical Activity Guidelines (PAG) and was meant to inquire if one engages in a structured form of physical activity (PA) or other known exercise training (ET). While PA can include exercise, it is not considered ET unless it follows a regimented routine. The ET guidelines developed by the United States Department of Health and Human Services have been the same since 2018 due to its compelling evidence of mortality benefits [1,2,3]. However, according to the World Health Organization (WHO), as many as one in three women and one in four men fail to engage in sufficient PA to maintain or improve health [4]. Despite patient interest in ET to improve health and provider recognition of PA benefits, some providers feel unqualified to prescribe ET, and some assume patient disinterest. In conjunction, patients do emphasize the need for enhanced professional support to address any potential barriers to ET participation [5]. As such, properly trained healthcare providers, as well as well-informed patient populations, are essential for an effective ET program. Therefore, the intent of this review is to review the current ET guidelines, the impact of physical fitness and how it relates to mortality benefits, and the molecular adaptations that occur with sufficient ET and to offer insight on the future direction and sustainability of ET programs.

## 2. Discussion

### 2.1. ET Guidelines

The 2018 PAG recommends at least 150 min of aerobic ET (aET) per week (min/week) or at least 75 min/week of vigorous-intensity aET [1]. The most recent aET guideline from the 2020 European Society of Cardiology is similar to PAG, although it encourages a longer aET duration of 300 min/week to maximize health benefits. Significantly deconditioned patients are encouraged to start with shorter ET durations [6,7]. For non-aerobic or resistance ET (rET), the recommendation is to attend at least two to three weightlifting sessions per week. One session should work on multiple muscle groups with 2 to 3 sets of 8 to 12 isometric contractions or lifts at moderate to vigorous relative intensity [1,6,8]. Remarkably, rET offers greater flexibility for individuals struggling to allocate enough time for aET, as notable benefits in terms of mortality can still be observed even with sessions lasting less than an hour (Table 1) [9].

### 2.2. Cardiorespiratory Fitness (CRF) and Mortality

It is important to know that effective aET regimens are measured by an increase in CRF. More fitness is associated with significant reductions in cardiovascular (CV) and all-cause mortalities, and in those with lower CRF levels, the risk of death is almost double, with even more pronounced mortality rates in those with concomitant metabolic syndrome (MetS) [11,12,13]. Some have studies revealed that men and women who belong to the 98th percentile of fitness generally outlive those who belong to the lower 20th percentile—by as much as 6 and 6.7 years, respectively—also showing that elite athletes tend to live longer than the general population [14,15]. While there are concerns of adverse cardiac issues with high-volume and high-intensity ET, these are only seen in those with low CRF who undergo extremely overzealous ET (exercising at a high intensity and volume far beyond recommended guidelines, often without adequate rest or recovery) [16,17]. Although discussed later, high-intensity ET appears to be safe for CAD and HF but could cause adverse outcomes in those with arrhythmia. Therefore, when unsure of current fitness level or comorbidities, it is important to train at moderate ET intensities.

There are two ways to estimate ET intensity level. The first is through familiarization with certain movements classified by their respective metabolic equivalents (METs) level. The American College of Sports Medicine (ACSM) provides an extensive compendium of PAs with corresponding METs levels. Moderate intensity is typically between 3 and 6 METs, whereas vigorous intensity is METs > 6 [18]. The second and gold-standard method to measure METs is by direct measurement of the maximum oxygen uptake (VO2max) an individual can utilize during intense ET. Importantly, VO2max indicates an individual’s aerobic capacity, which is the hallmark for CRF [19].

### 2.3. Muscular Strength (MusS) and Mortality

Another measure of physical fitness is MusS, which is achieved through rET. Increasing MusS can significantly lower mortality risks, even after accounting for the benefits of improved CRF and body composition. MusS is also associated with decreased risks of age-related weight gain, hypertension (HTN), and MetS and plays a role in improving insulin sensitivity and certain cardiometabolic profiles [20]. A cohort study by Liu and colleagues revealed that engaging in rET one to three times weekly (all 3 groups with *p* ≤ 0.05), with each session lasting less than an hour, significantly lowered the risk of total CV disease (CVD) events (morbidity and mortality combined) by 70%, irrespective of participation in aET or improvement in CRF. The findings also indicated that additional rET beyond this frequency does not enhance survival benefit [9].

### 2.4. Muscle Mass and Mortality

Measures of physical fitness like CRF and MusS do not only matter for longevity but also for appearances. Muscle size has been shown to be intimately related to mortality risk. A 2022 systematic review and meta-analysis published in *Karger’s Gerontology* analyzed the relationship between low muscle mass and sarcopenia across different population groups. It was shown that adults with sarcopenia had twice the risk of mortality compared to controls (HR = 2; 95% CI: 1.71–2.34) [21]. In 2023, a cohort study on Chinese community-dwelling adults by Xiong et al. showed that the 7-year mortality odds ratio (OR) for those with sarcopenia was (OR = 1.41; 95% CI: 1.06–1.87; *p* < 0.05). The risk was more than double in those with severe sarcopenia, rising to as much as 3 to 5 times the likelihood in the 60 to 80 age group and >80 age group, respectively [22]. Therefore, in addition to improving CRF and MusS, building muscle mass and addressing obesity are very important elements of improving health beyond the benefits of esthetics.

### 2.5. The Case of the Unfit

Despite the aforementioned knowledge from various high-powered studies, CVD remains one of the leading causes of mortality globally per the WHO [23]. Recently, the American College of Cardiology published a large cohort study on behalf of Kokkinos et al., which revealed breakthrough data indicating that the single greatest risk for all-cause mortality across age, race, and spectrum is being physically unfit. Major traditional CV risk factors like dyslipidemia (DLD), diabetes mellitus (DM), HTN, and chronic kidney disease (CKD) can be favorably modified by increasing CRF levels. For instance, the risk of death was further amplified in those who had traditional CV comorbidities but had poor fitness (HR = 4.09; 95% CI: 3.94–4.24; *p* < 0.001), whereas in those who were very fit and had one pre-existing traditional CV comorbidity, the risk of mortality did not increase. While smoking is also a major CV risk factor, the risk from smoking can be modified with simple cessation. Therefore, significant lifestyle modification by increasing PA, along with smoking cessation, can significantly attenuate the risk of death from CVD (Table 2) [24,25,26].

### 2.6. DLD and ET

It is practically established that ET attenuates atherogenic lipoproteins like low-density lipoprotein cholesterol (LDL-C) while simultaneously improving cardioprotective lipoproteins like high-density lipoprotein cholesterol (HDL-C). A study conducted in 2007 by Halverstadt et al. (n = 100; sedentary 50–75 years old) revealed that total cholesterol (TC), triglycerides (TG), and LDL-C decreased with ET (*p* < 0.001, *p* < 0.0001, and *p* < 0.0001, respectively), whereas HDL-C subfractions (HDL2-C and HDL3-C) increased significantly (*p* < 0.02 and *p* < 0.01, respectively) [27]. Leon and Sanchez conducted a review of 51 studies (28 were randomized controlled trials or RCTs) that analyzed the effect of more than 2 weeks of moderate to vigorous aET on lipids. The study showed that, on average, HDL-C increased by 4.6% from baseline, whereas LDL-C and TG decreased by 5% and 3.7%, respectively. However, lipoprotein response was largely inconsistent with aET, except for HLD-C, which appeared to be consistently responsive [28]. Tambalis et al. later reviewed more (n = 84) trials, wherein 58 studies were RCTs. The investigators analyzed the lipid responses from three separate arms stratified by ET modalities, namely, aET, rET, and aET plus rET. Results revealed that specific lipoprotein levels were more likely to respond to certain modes of ET. For example, HDL-C consistently increases with aET compared to other lipoproteins, while LDL-C was more consistently attenuated by rET. Concordantly, combining aET and rET consistently yielded improvements in both LDL-C and HDL-C levels [29]. These findings suggest that while aET and rET can be independently beneficial in the treatment of DLD, larger benefits can be seen when applying both aET and rET.

Some studies have demonstrated that HDL-C levels of about 73 mg/dL and 93 mg/dL are associated with the lowest risk of all-cause mortality in men and women, respectively. However, the mortality benefits from HDL-C decrease at lower and higher extremes, mimicking an inverted U shape. These facts are particularly important to know in order to help clinicians decide whether to begin pharmacologic therapy when HDL-C levels remain low, even with ET, or when to discontinue drugs when ideal HDL-C levels have been attained [10,13,30,31,32]. The same applies to LDL-C, for which low and high extremes were associated with a higher risk of all-cause mortality. Unlike HDL-C, the optimal LDL-C level still needs to be determined by future studies [33].

Recently, HDL-C particle characteristics have been a topic of interest. Due to variations in age, race, gender, and other confounders, there can be variations and inconsistencies in ET-related changes in total HDL-C levels. However, some studies have been able to demonstrate that the favorable effects of ET as it relates to HDL-C can, likewise, be attributed to increases in HDL-C subfractions (HDL2-C and HDL3-C), which possess significant antiatherogenic properties. Thus, not only can quantitative alterations in serum HDL-C levels be seen with ET but also qualitative improvements in HLD-C particle maturation, size, composition, and functionality [34,35,36]. Alteration in LDL-C particle size is also important during ET. While conventional recommendations from the Heart Protection Study and the Pravastatin or Atorvastatin Evaluation and Infection Therapy trial (PROVE-IT/TIMI-22) emphasize targeting LDL-C levels, studies have shown that this can only usually be achieved with weight loss.

However, CV mortality benefits can certainly be seen even without significant weight loss or quantitative attenuation of LDL-C. Some studies have revealed that moderate to vigorous aET can reduce smaller and more atherogenic lipoproteins like very low- or intermediate-density lipoproteins while simultaneously increasing LDL-C particle size; therefore, larger LDL-Cs would likely have less propensity to traverse the endothelium. These findings can explain why improved CV outcomes can still be seen in fit populations, even if LDL-C levels and body weight have not significantly changed [37,38,39,40,41,42,43,44,45].

The main driving point is that relying solely on weight loss overlooks the underlying mechanisms of metabolic diseases. Many patients with type II diabetes, metabolic syndrome, and other conditions can achieve significant health improvements or even remission through appropriate therapy, exercise, and diet, without necessarily losing weight. While this is acknowledged in relation to cardiovascular mortality, it applies broadly to other conditions as well.

### 2.7. Glucose Regulation and ET

The expression of GLUT4 glucose transporters in cellular membranes promotes the uptake of glucose into adipose and muscle tissues [46]. During ET (both aET and rET) and insulin exposure, the AMPK (AMP-activated protein kinase) signaling system is activated and causes an increase in GLUT4 translocation to the cellular membrane, causing enhanced glucose regulation [47]. 

Therefore, during states of insulin resistance or low PA levels, there is a decrease in GLUT4 receptor expression [48]. Individuals who have DM are a population of particular interest, since they naturally have lower insulin sensitivity [49]. Per the AHA, although PA can lead to an immediate rise in GLUT4 mRNA expression, the effects are only transient, and continuous ET is needed in order to sustain GLUT4 protein in the longer term. PA can transiently increase insulin sensitivity and, thereby, lower plasma glucose for up to 24 h or more, but with continuous ET, cumulative increases in GLUT4 mRNA and protein expression in muscle cells can be seen. This process ultimately enhances glucose regulation and improves insulin sensitivity [50,51,52].

It is also known that rET prevents metabolic dysfunction in those with obesity and DM [53]. Besides improving MusS, rET can increase muscle mass, which can increase insulin sensitivity and the metabolic rate [54]. Although the improvement of glycemic control and insulin sensitivity can be seen in both aET and rET, there are some data suggesting that rET is more effective [55,56]. In summary, those who perform ET regularly benefit from enhanced glucose control from the effects of increased insulin signaling, increased muscle mass, and improved mitochondrial expression during ET.

### 2.8. Renal Adaptations with ET

Even though there is not a single, specific groundbreaking study or finding regarding the effects of moderate ET on the progression of CKD, there is still an increasing body of evidence suggesting that PA is associated with a decelerated rate of decline in renal function in CKD patients based on many observational studies. In a systematic review conducted by MacKinnon et al. encompassing 29 studies involving CKD patients who were not undergoing dialysis, as well as individuals with kidney transplants, it was observed that decreased levels of PA were linked to increased mortality rates [57]. On the other hand, other observational studies including small RCTs have shown that higher PA levels are linked to lower all-cause mortality [5,58,59,60], although more studies are needed to establish causality.

One concern is the safety of ET in those who have renal dysfunction. However, in a recent RCT by Weiner et al. (n = 99; mean age 68 years), compliance with a 1-year combined aET and rET program was proven to be safe and was associated with improvement in overall physical functioning [61]. Likewise, older RCTs demonstrated improvement in physical functioning, using gait speed and VO2max as measures [58,62]. However, renal transplantation recipients should exercise with caution, especially when engaging in prolonged exercise like marathons or ultramarathons, due to the risk of pre-renal damage from dehydration [63]. Ultimately, the benefits of regular ET outweigh the risks associated with sedentary behavior in CKD patients. This is important, since the majority of patients who have moderate CKD do not usually survive to the point of reaching end-stage renal disease [62,64].

There are many ways ET can help CKD patients. As mentioned earlier, ET has been shown to enhance insulin sensitivity, which mitigates kidney damage associated with DM. Another advantage of ET is that it can induce anti-inflammatory properties, which can help mitigate additional renal damage, especially in individuals with diabetic nephropathy. Aldarh and El-Kader studied 80 obese patients with a mean type 2 DM (T2DM) chronicity of 12.53 ± 2.64 years. After being divided into 2 groups (aET and no aET), findings indicated that aET reduces systemic inflammation by lowering pro-inflammatory cytokines such as interleukin-6 and tumor necrosis factor-alpha (TNF-α). In T2DM patients, Bote et al. were able to observe a reduction in levels of IL-8, which is the primary inducer of chemotaxis during inflammation, after patients completed 32 weeks of aquatic ET [65]. Hong et al. also observed that inflammatory processes were lower in those who were more fit or had higher CRF [66]. aET also exerts anti-inflammatory effects by increasing cytokines like interleukin-10 (IL-10), glutathione peroxidase, and glutathione [67].

A recent meta-analysis (n = 1603) from 2023 revealed that rET has more attenuating capabilities compared to aET or combined ET modalities, with statistically significant results reports. It was concluded that rET may be particularly more effective in reducing levels of pro-inflammatory markers such as TNF-α and C-reactive protein while simultaneously enhancing the levels of anti-inflammatory cytokine IL-10 [68,69]. These findings suggest that rET may be more beneficial when it comes to inflammatory modulation.

Arazi et al. found that ET can also increase levels of L-arginine, which is a precursor of nitric oxide (NO). This causes an activation of endothelial NO synthase, which can relax renal vasculature to improve renal blood flow. ET was also observed to simultaneously prevent NO degradation by decreasing circulating reactive oxygen species (ROS). NO can also help decrease kidney stone risk and lower blood pressure (BP) in those with concomitant systemic HTN, which is one of the leading causes of kidney disease [70]. Another interesting effect of ET on the renal system is that it can prevent anemia, which is the most common trigger for type 2 acute myocardial infarction (MI) and results from oxygen–tissue exchange mismatch in times of increased cardiac demand [71].

Anemia is also associated with an increased risk of poor outcomes in heart failure (HF), coronary artery disease (CAD), atrial fibrillation (AF), and stroke [72]. Anemia can be a result of poor red blood cell production due to poor erythropoietin (EPO) production. In a study by Corrêa et al., individual athletes (n = 39) with an average age of 23.24 years who reported high levels of PA in the International Physical Activity Questionnaire were found to have higher concentrations of EPO and erythroferrone levels compared to the control group (*p =* 0.001 and *p =* 0.0034, respectively) [73,74]. However, more studies are needed in order to determine the precise mechanism of how ET induces an increase in EPO production.

### 2.9. Vascular Adaptations with ET

As mentioned in the previous section in the case of HTN, it is known that aET effectively decreases BP. As per the 2004 guidelines from the ACSM, engaging in aET typically decreases systolic BP and diastolic BP by as much as 5 to 7 mmHg, regardless of intensity. These effects usually only last for about 22 h, whereas rET induces a shorter BP-lowering duration [75,76]. These findings highlight the importance of regular ET to sustain the BP-lowering benefits. Based on the aforementioned findings, the reduction in BP is attributable to a decrease in norepinephrine (NE), which results in attenuated sympathetic tone. Downstream vasoactive hormones such as angiotensin II, adenosine, and endothelin decrease when NE is lower. On the other hand, ET increases hormones like NO and prostaglandins to relax vascular tone [77]. In addition, endothelial dysfunction itself can lead to HTN by increasing oxidative stress from ROS, which can lead to a significant reduction in NO bioavailability [78,79]. Reduced ROS production effects can be achieved through aET, which can effectively diminish nicotinamide adenine dinucleotide phosphate (NADPH) oxidase activity. aET has also been found to decrease left ventricular (LV) mass and arterial wall stiffness [78]. Thus, the interplay of these hormonal effects contributes to reductions in CV events resulting from chronic HTN [80].

### 2.10. Heart Failure and ET

There have been several large RCTs showing reduced HF hospitalizations and increased quality of life (QoL) in association with ET [81,82]. There are also convincing data from 2012 showing that long-term (≥6 months) aET can reverse LV remodeling. However, rET, whether alone or in combination with aET, neither improved nor worsened LV remodeling [83]. A very recent study published in February 2024 by Hsu et al. revealed that High-Intensity Interval Training (HIIT) improves all-cause mortality in patients with varying HF severities, as in those with reduced ejection fraction (HFrEF), HF with mid-range EF (HFmrEF), and HF with preserved EF (HFpEF). In the study, the participants were subdivided according to their corresponding LVEF (<40%, ≥40% and <50%, and ≥50%). Among all included participants, the 10-year survival was higher (*p* = 0.015) for those who underwent HIIT (80.3%) than for participants receiving guideline-directed medical therapy (68.6%). These mortality benefits were linked to various factors, like a reduction in LV diameter during end-diastole and an increase in VO2max. The study also underscores the safety of vigorous ET, even for patients with stable HFmrEF and HFpEF who are not in decompensated HF [84].

ET via early cardiac rehabilitation (CR) benefits HF patients after acute MI (AMI). In a study conducted by Cai et al., it was observed that 2 weeks of CR significantly reduced cardiogenic deaths in those with HFrEF and decreased hospitalization rates in those with HFmrEF. Data showed that the Non-CR group had a higher incidence of death (31.3% vs. 0.00%, *p* = 0.002) and a higher incidence of re-hospitalization (22.1% vs. 3.6%, *p* = 0.008) compared to the CR group. In this study, early CR helped patients develop and maintain long-term home-based exercise habits, leading to better cardiac remodeling and myocardial perfusion [85].

Of note, cardiac muscle growth can be categorized as either concentric or eccentric using the relative wall thickness, although cardiac growth can be expected in either ET-induced or pathologically induced mechanisms. Pathological growth is marked by features like interstitial and perivascular fibrosis and myocyte disarray, which are usually seen in hypertrophic cardiomyopathy. Fibrosis and cardiac muscle disarray are not seen with chronic ET. In genetic mouse models, the PI3K(p110α)-Akt signaling pathway, insulin-like growth factor-1, and insulin mediate physiologic growth and counter pathologic hypertrophy with chronic ET. This groundbreaking research emphasizes the potential of ET to improve long-term outcomes in individuals with HF across a spectrum of EF readings, offering hope and opportunities for improved patient care and management [84,86]. In rat models with aortic stenosis, it was noted that aET can restore activity from cardiac impairment in failing hearts and the expression of proteins that are responsible for mediating calcium (Ca^2+^) handling and flow. In the study, rats subjected to aET showed improved cardiac muscle responses in post-rest contraction tests, including optimized myofilament responsiveness to Ca^2+^ [87]. Therefore, future studies would be helpful in validating the cardio-histological differences caused by ET vs. pathologically induced mechanisms in order to allow providers to reassure patients on the effectiveness and safety of ET for those with stable HF.

### 2.11. Atrial Fibrillation and ET

Newer studies have also shown that structured ET is now being considered as part of the comprehensive management of AF. In the ACTIVE-AF trial, which involved 120 participants with either paroxysmal AF (PAF) or symptomatic AF, participation in a 6-month aET program that was both supervised and implemented in home-based settings, led to significant reduction of AF recurrence and symptom severity compared to the standard of care. At the end of the freedom from AF analysis, 40% of ET participants were free from AF, whereas only 20% were free from AF in the standard of care group. The risk of recurrence was 0.50 (95% CI: 0.33–0.78; *p* = 0.002), implying a 50% reduction in the chance of AF recurrence. In addition, symptom severity analysis showed that participants who completed the 6-month aET program had significantly lower symptom severities compared to participants who did not exercise. Outcomes were not statistically different when the program was extended to 12 months (*p* = 0.041) [88,89].

Some of the beneficial electrical adaptations with ET include enhanced vagal tone and improved autonomic balance, which stabilize heart rhythm and reduce arrhythmia risk. However, intensive and prolonged ET can also lead to harmful changes like increased myocardial fibrosis and scarring, which disrupt normal Na^+^, Ca^2^⁺, and K⁺ ion channel function. These changes could interfere with normal electrical pathways. Therefore, intense ET may be potentially arrhythmogenic in those with AF. These changes underscore the importance of individualized exercise prescriptions to maximize benefits without concomitantly increasing risk [90].

The Cologne ExAfib Trial is a Phase I RCT examining the feasibility and safety of different ET modalities in those with PAF. As it compares moderate-intensity aET, HIIT, rET, and standard of care over a 12-week period, the study also aims to explore many secondary ET outcomes in those with AF, such as overall physical function; AF burden; QoL; and degree of inflammation, including impact on cardiac muscle morphology and function [91]. With promising studies like this in development, there is potential to establish more precise ET guidelines specific to AF patients, since ET may be both beneficial and harmful in this population.

### 2.12. Malignancy and ET

There is strong evidence indicating that regular PA can attenuate certain types of cancers. A pooled analysis conducted in 2016 involving 1.44 million subjects showed that moderate to vigorous leisure-time PA was associated with a decreased risk for 13 out of 26 types of cancers, namely esophageal, liver, lung, renal, gastric, endometrial, myeloid leukemia, myeloma, colon, rectal, head and neck, bladder, and breast cancers. These relationships were apparent irrespective of body mass index (BMI), except for certain obesity-related cancers, where BMI played a more significant role, and smoking background, underscoring its generalizability across different population groups [92,93,94]. A 2019 study published by the American Cancer Society in partnership with the National Cancer Institute and the Harvard T.H. Chan School of Public Health discovered that achieving the recommended levels of 2.5 to 5 h of moderate PA per week was associated with lower risk of developing seven types of cancers, namely colon, breast, endometrial, kidney, multiple myeloma, liver cancer, and non-Hodgkin lymphoma. In addition, vigorous ET of about 1.25 to 2.5 h per week was associated with a reduction in cancer risk, with more pronounced benefits associated with longer-duration ET [95].

With respect to the aforementioned relationships, it is important to know that malignancy and associated cancer treatments are closely associated with CVD risk. In 2022, Wang et al. noted that cancer-related chronic inflammation can exacerbate the progression of atherosclerosis and CVD [96]. Another way cancer and CVD share similarity is through the clonal hematopoiesis of indeterminate potential (CHIP) mutation. CHIP mutations cause an increased risk of somatic mutations in hematopoietic stem cells, which can lead to leukemia and a simultaneous increase in atherogenesis and coronary calcification, doubling the risk of CAD. CHIP mutations are usually seen in aging populations, increasing over time, whereas they are normally absent in children. Thus, prognosis is very poor in cancer patients with CHIP mutations, since CAD is almost always a concurrent issue [97,98,99,100,101,102]. CHIP mutations can increase macrophage-mediated inflammatory processes, and interestingly, the inflammation itself can lead to further somatic mutations, thereby exacerbating the development of cancer and CAD [96]. Furthermore, CVD shares many of the major risk factors seen in cancer, such as natural aging, poor diet, and smoking, with great emphasis on physical inactivity [103].

**Figure 1:**
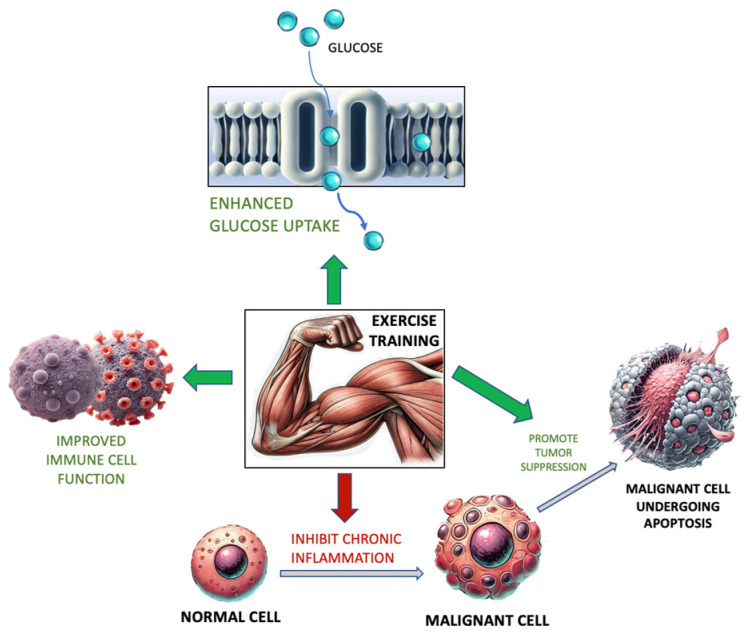
Exercise training and its relationship with glucose uptake, immune cell function, inflammation, and tumor cell activity. Adapted from [104].

In 2023, Spanoudaki et al. suggested that cancer risk and progression may be mediated by ET, which can enhance hormonal balance, insulin sensitivity, and immune cell function and mitigate many inflammatory and oxidative processes. These processes, in tandem, aid in the elimination of cancerous cells. (Figure 1) [104]. When analyzing these data, it is important to remain vigilant of the shared risk factors between CVD and malignancy so as to emphasize the utility of ET in these domains.

### 2.13. Future Directions and Diverse Modalities of Personalized ET Approaches

In addition to establishing universally recognized guidelines for ET and shedding light on the molecular changes that confer both physiological and CV benefits, it is crucial to examine the potential ways to increase ET adherence. With the advent of advanced technology and artificial intelligence, wearable devices such as fitness trackers, smart watches, and virtual reality (VR) are increasingly being utilized to support ET programs. The growing popularity of smart watches can be ascribed to their multifunctional capability to monitor various health metrics, including cardiac rhythm/rate, oxygen levels, and even activity levels [105]. However, recent literature has highlighted some inconsistencies in the reliability and validity of the various metrics captured by the aforementioned wearables. This is mainly due to the fact that the algorithms used in some of these technologies are not often standardized [106]. For instance, many wearable devices do not have the capability to record data over 24 h but instead rely on short-term data acquisition. Many factors, such as gender, current state of health, and age, can also influence metrics like heart rate variability (HRV). A 2021 study conducted by Hinde et al. showed that the validity and reliability of HRV readings decreased with increasing ET intensities [107,108]. In 2019, Passler et al. measured VO2max with wrist-worn activity trackers such as the Garmin Forerunner 920XT (Garmin Ltd., Olathe, KS, USA), which significantly underestimated maximum oxygen uptake, whereas other models like the Garmin Vivosmart HR overestimated metrics like energy expenditure (*p* = 0.027 and *p* = 0.023, respectively) [109]. As such, it seems that these commercially available devices still lack accuracy in measuring ET intensities and CRF, making them not totally reliable in athletic and clinical settings. Nonetheless, these devices are not completely without utility and can still be used to estimate PA and fitness levels. In recent times, VR has also progressed beyond its initial gaming and entertainment boundaries, now emerging as a versatile tool that can be applied across various industries. Its most powerful feature is the creation of an immersive and secure environment where various tasks can be practiced and honed, offering a degree of realism that is both convincing and applicable to real-life scenarios. VR headsets are no longer exclusive to the gaming industry; they have proven their worth in making ET more effective by simulating engaging environments. This technology is particularly beneficial for those facing health challenges or logistical issues or who travel often, providing access to diverse classes of lifestyles. By incorporating game-like elements into ET, VR effectively engages users while simultaneously helping them stay motivated and on task with their fitness goals [110]. A study conducted by Segura-Ortí and García-Testal in 2019 consistently indicated that VR can safely boost PA to a greater extent than traditional ET methods in patients undergoing hemodialysis [111].

Although ET is widely recognized as the mainstay non-pharmacologic treatment for CAD, with its efficacy substantiated by a Cochrane review in 2021, the current guidelines primarily emphasize moderate-intensity continuous training (MICT). These types of exercises are usually longer in duration, which can pose significant challenges for less aerobically conditioned individuals like those with CAD [112]. Nonetheless, there are emerging programs that take these factors into consideration, such as HIIT programs, due to the nature of their short sessions and efficiency [113]. HIIT is increasingly recognized as being able to increase CRF and even QoL. Weston et al.’s meta-analysis of more than 200 participants across 10 studies revealed that low-volume HIIT significantly amplifies CRF levels. These improvements were observed across a diverse group encompassing adolescents, healthy adults, individuals with obesity, cancer patients, and those suffering from metabolic syndrome. Findings revealed that HIIT nearly doubled the CRF (measured in VO2max) level when compared to those who underwent traditional MICT (19.4% vs. 10.3%, *p* < 0.001) [114]. A recent study conducted by Kristiansen et al. showed that HIIT significantly increased VO2max by 2.5 mL/kg/min (95% CI: 2.1–3.0, *p* < 0.001) compared to standard of care, which increased VO2max by only 0.2 mL/kg/min (95% CI: 0.2–0.6, *p* < 0.001). Another study showed that after 12 weeks of HIIT, QoL improved in CAD patients when compared to usual care but to a lesser magnitude (*p* = 0.05). This further validates the applicability of high-intensity regimens like HIIT for frail populations. HIIT is a safe and effective alternative for those who have CAD and intend to increase CRF levels [115,116]. Increases in CRF were found to result from increases in stroke volume, cardiac output, red blood cell volume, and capillary density, which collectively improve overall oxygen-carrying capacity. Although safe in CAD patients, these ET programs may not be suitable for patients with significant physical injuries, disabilities, or limitations with respect to high-impact activities [117]. One popular variant of HIIT is CrossFit, which has been the most rapidly expanding category of vigorous ET globally since its establishment by Greg Glassman in 1996 in the United States. Initially developed for military strength and conditioning, CrossFit has gained widespread popularity among civilians over the years due to its effectiveness in enhancing VO2max, MusS, and body composition [118,119]. CrossFit achieves these benefits by optimizing physical functional competence through the activation of the body’s three primary metabolic pathways, namely (1) the phosphagen pathway (ATP-phosphocreatine system) for immediate bursts of high-intensity effort, (2) the glycolytic (anaerobic glycolysis) pathway for prolonged high-intensity effort beyond 6 to 10 s, and (3) the oxidative (aerobic) pathway for continuous energy production after several minutes of activity. A 2022 publication from the Cleveland Clinic outlines that training the phosphagen system requires brief, high-intensity exercise intervals coupled with comparatively longer rest periods, unlike the glycolytic system, which is trained through moderate-intensity activities lasting a few minutes, with shorter rests in between. Lastly, the oxidative (aerobic) pathway benefits from low-intensity exercises such as extended cycling or swimming sessions [120,121]. The key characteristic of CrossFit is the rapid and repetitive execution of high-intensity exercises with little to no rest between sets while activating the aforementioned metabolic pathways to improve functional fitness [122]. Although there are concerns regarding injuries, there have been several investigations showing that the prevalence of injuries related to CrossFit is similar to that in other sports disciplines, such as gymnastics, weightlifting, and powerlifting. Most injuries are likely to be observed in those who are new to the sport [123,124,125,126] and usually affect the shoulders more than other body parts [127]. In addition, higher injury rates are observed more often in popular team-based sports [128]. While these personalized ET programs are intended to increase motivational aspects, CrossFit has been one of the pioneers of increasing PA adherence due to the concept of community-driven fitness. Further findings indicate that increased ET frequency correlates with enhanced social functioning and improved body-image perceptions, while long-term involvement is inversely related to disordered eating behaviors [129,130]. Finally, fitness apps with additional tracking and/or social features are now integrated in various indoor equipment, wearables, and handheld devices, which can potentially advance our understanding of ET and accessibility. With various ET modalities such as HIIT emerging, we can further broaden ET interest to improve essential fitness parameters like CRF, MusS, and flexibility.

## 3. Conclusions

Even with robust data validating the effectiveness of the current ET guidelines in terms of morbidity and longevity, CVD prevalence continues to grow and, partly due to the underutilization of ET amidst a context of advanced technology and pharmaceuticals. It is important to understand how several CV issues can be effectively addressed by the molecular impact of increased PA. However, to improve lifestyle is a lot more challenging than simply prescribing an ET regimen or a pill. Previous misconceptions about vigorous ET should also be revisited because new research suggests that intense ET regimens like HIIT are not only effective but also safe for individuals with ongoing CV conditions (i.e., stable CAD and HF).

Incorporating ET into effective health management strategies involves not only setting global ET guidelines and expanding our knowledge on its molecular impacts on health but also finding more ways to improve ET compliance with the aid of advanced technology, as well as by utilizing tailored exercise programs to make ET more engaging and accessible, particularly for those with health challenges or logistical constraints. Leveraging both emerging research and innovative technology could help reshape societal norms on the issue of PA, ensuring that everyone can access the profound health benefits of a more active lifestyle, which is the single most effective pill of all.

## Figures and Tables

**Table 1:** Summarized exercise prescription for adults based on the 2018 Physical Activity Guidelines (PAG) for Americans, 2nd edition and the 2016 ESC Exercise Guideline. Adapted from [10].

Mode	
Aerobic ET	Brisk walking, running/jogging, swimming, bicycling, stair climbing, rowing, aerobic dancing, skiing
Resistance ET	Lifting weights, resistance/elastic bands, bodyweight exercises, heavy gardening, calisthenics
**Duration**	
Aerobic ET	150–300 min/week (moderate intensity); 75–150 min/week (vigorous intensity); Or an equivalent combination of moderate to vigorous intensity
Resistance ET	8 to 12 repetitions to fatigue; at least 1 set for all muscle groups; 60 to 80% intensity of single maximum repetition (70% in elderly)
**Frequency**	
Aerobic ET	Most days of the week (preferably 6–7 days/week)
Resistance ET	2–3 non-consecutive days/week
**Intensity**	
Aerobic ET	Moderate to vigorous intensity
Resistance ET	Moderate to vigorous intensity

**Table 2:** Relative mortality risk associated with traditional CV risk factors and fitness levels. Adapted from [26].

Variable	Hazard Ratio (HR)	*p*-Value
Fitness Levels		
Least	4.09 (3.94 to 4.24)	<0.001
Low	2.88 (2.78 to 2.99)	<0.001
Moderate	2.13 (2.05 to 2.21)	<0.001
Fit	1.66 (1.60 to 1.73)	<0.001
High	1.39 (1.34 to 1.45)	<0.001
Comorbidities		
Chronic kidney disease	1.49 (1.46 to 1.52)	<0.001
Smoking	1.40 (1.39 to 1.42)	<0.001
Diabetes	1.34 (1.33 to 1.36)	<0.001
Atrial fibrillation	1.34 (1.31 to 1.36)	<0.001
Cancer (all)	1.33 (1.30 to 1.35)	<0.001
CV Disease	1.28 (1.27 to 1.29)	<0.001
Hypertension	1.14 (1.13 to 1.16)	<0.001
Age	1.06 (1.06 to 1.06)	<0.001
Body Mass Index	0.98 (0.97 to 0.98)	<0.001
